# Chronic Articular Inflammations

**Published:** 1846-12

**Authors:** 


*Chronic Articular Inflammations.*—M. Biéckly prefers the following formula:

Nitrate of silver, ℈iv.
Water, sufficient to dissolve this salt.
Lard, ℈xxxii.
Mix.

Frictions are to be made twice a day, with 4 or 5 scruples each time. The third or fourth day the skin will have the aspect of shining black polished leather. These frictions produce no pain nor tegumentary irritation, if we except some few vesicles. The treatment ought to be stopped now for a few days, until the black epiderum peals off, and the skin resumes its normal color. Under this ointment, deep articular alterations are modified; the heat and pain subside, the effusion is absorbed and the swelling dissipated. M. B. thinks the preparation is absorbed.—*S. Med. & Surg. Jour.*

